# Assessing Effect of Rootstock Micropropagation on Field Performance of Grafted Peach Varieties by Fitting Mixed-Effects Models: A Longitudinal Study

**DOI:** 10.3390/plants12030674

**Published:** 2023-02-03

**Authors:** Juan A. Marín, Elena García, Pilar Lorente, Pilar Andreu, Arancha Arbeloa

**Affiliations:** Pomology Department, Estación Experimental de Aula Dei CSIC, Av. Montañana 1005, 50059 Zaragoza, Spain

**Keywords:** *Prunus persica*, *Prunus dulcis* × *P. persica*, *Prunus insititia*, Adafuel, Adesoto 101, multilevel model, subject level randomization, variety crossed effect, linear mixed-effects model

## Abstract

Rootstock micropropagation has been extensively used as an alternative to propagation by cuttings. Although studies have recently been conducted on other species, no conclusive reports have been published on the effect of rootstock micropropagation on the field performance of fruit trees. Here, we present the results of a five-year study of peach varieties grafted on two rootstocks (Adesoto 101 and Adafuel), either micropropagated or propagated by cuttings, to ascertain the effect of the rootstock propagation method on field performance. Fruit trees are woody plants with a long life cycle; so, to reduce the influence of environmental or cultural factors on the agronomical results, studies need to last for several years, in which data are obtained from the same individuals over time (longitudinal data). This hinders the analysis because these data lack independence. In contrast with a more traditional approach with data aggregation and repeated-measures ANOVA analysis, in this study, we used linear mixed-effects models to control the variance associated with random factors without data aggregation. The growth of the fruit trees did not appreciably differ between the rootstock propagation methods, neither in the flowering period nor in the yield. The models constructed for different parameters of the field performance (trunk cross-sectional area (TCSA), cumulative yield, cumulative yield efficiency, and cumulative crop load) showed a very good fit (R^2^ > 0.97), allowing the conclusion that the rootstock propagation method did not affect the field performance of fruit trees in this study.

## 1. Introduction

Rootstock micropropagation has been extensively used as an alternative to propagation by cuttings because of its advantages to the nursery industry. Micropropagation enables, among other things, mass propagation, plant homogeneity, propagation of difficult-to-root clones, and plant health control. Micropropagated self-rooted varieties show changes that affect flowering delays, mainly in the first year of growth, as observed in forest species [1], wild cherry [2] and blueberry [3], or excessive branching, as noted in wild cherry [2], blueberry [3], and apple [4]. These effects may be related to the rejuvenation of the plant due to the action of plant growth regulators during tissue culture [5,6]. Notably, these adverse effects of micropropagation do not occur with fruit trees when micropropagated rootstocks are grafted with commercial varieties. However, only a few preliminary reports have been published on the effects of micropropagation on the field performance of different fruit tree rootstocks: peach [7], sour cherry [8], and apple [8,9]. Self-rooted varieties have been compared with the corresponding grafted ones, but differing results have been reported. Micropropagated self-rooted Chandler walnut was more vigorous and homogeneous than grafted trees [10]; in peach, whereas self-rooted trees were more vigorous than the grafted ones, yield did not differ during the second crop [11].

Agronomical studies on fruit trees, which are woody plants with a long life cycle, need to be conducted over several years in which data are obtained from the same individuals over time (longitudinal data) to reduce the effect of environmental and cultural factors, which are difficult to control, on the response to the applied treatments. Longitudinal data collection is a special case of repeated measures in which variables are measured over time. Data collected from the same individual are likely correlated; thus, they cannot be considered independent. Therefore, the use of traditional methods of analysis based on ANOVA is not a valid option, unless we process some of the information through data aggregation [12]. Researchers in other fields have solved the problem of a lack of independence in longitudinal data by using mixed-effects models [13,14,15,16]. These models combine fixed effects (i.e., those we can control) with random effects, for example, those related to individual subject variability, either alone or in combination with other factors. In this study, we used linear mixed-effects models to control variance associated with random factors without data aggregation instead of the more traditional approach with data aggregation and repeated-measures ANOVA. Fruit tree data show notable variability year by year, because fruit trees are affected by changing environmental and cultural conditions. Additionally, this generated noise can be easily reduced by using cumulative data. Thus, cumulative data values linearly increase over time, enabling their modeling through linear models.

A linear model describes a response variable as a combination of predictor variables and can be expressed as:

Response variable = intercept + slope ∗ predictor variables,

where intercept is the baseline relationship between response and predictor variables, and the slope indicates the strength of this relationship.

Linear mixed-effects models are constructed by adding randomly varying values to both the intercept and slope, originating from fixed effects.

In this study, we aimed to answer two questions:

1. Do linear mixed-effects models produce a satisfactory goodness of fit in which predicted values accurately match with the observed values? If so, then:
2. Does the rootstock propagation method (micropropagation or cuttings) affect the field performance of grafted peach trees?

## 2. Results

Tree growth did not differ between those grafted on micropropagated rootstocks and those grafted on cuttings. Only Baby Gold 5 trees, grafted on Adafuel propagated by cuttings, showed increased vigor compared with those grafted on micropropagated rootstocks. These vigor differences were maintained throughout this study, but did not affect yield. In addition, trees showed no differences in the blooming period, yield, or the ripening date. The varieties have different ripening dates, which, in our locality, were late June for Super Crimson Gold, early July for NJC97, mid-July for Catherine, and late July for Baby Gold 5.

### 2.1. Model Construction

To construct our mixed-effects models, we fit both fixed and random effects in a two- step process [17]: First, we identified the random effects that best fit the data, without including fixed effects, obtaining a null model that was fit to the maximal likelihood estimate. Second, we fit the fixed terms of the model.

### 2.2. Random Effects

**Figure 1:**
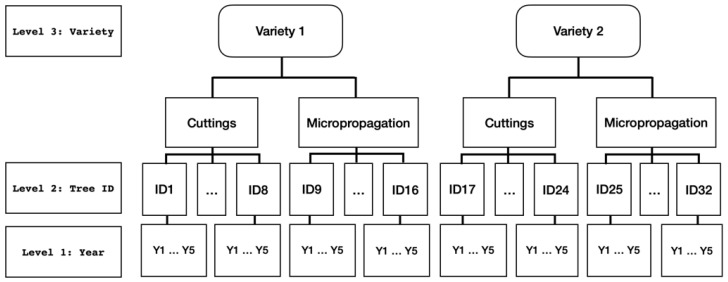
Experimental model: three-level model with subject-level randomization and variety crossed effect.

The random-effects structure reflects our understanding of where to expect variance, and how data interact with that variance. We started from our experimental model (Figure 1), which was a multilevel (three levels) model: subject-level randomization with a variety crossed effect.

Following Magnusson [18], variety was a crossed effect; that is, in a parallel group design, both varieties received both treatments (propagation by cuttings and micropropagation). Because this was a randomized trial, in this design, we applied subject-level randomization and, at the variety level, we included random effects for time, treatment, and the time × treatment interaction. Each subject–variety interaction had random intercepts and slopes as influenced by time applied to it, and the intercepts and slopes of the variety in the model could be affected by random factors (different baselines or different average effects per variety), whereas slopes could be affected by treatment, time, or the interaction between time and treatment.

The saturated model included the terms listed in Table 1 (in R notation):

This is a saturated model, which could have shown a high correlation among some terms that had to be removed to improve goodness of fit; therefore, a desirable, more parsimonious model was developed.

After removing unnecessary or redundant terms, the null models have reduced numbers of parameters that need to be estimated, increasing goodness of fit. The Akaike information criterion (AIC) indicates a better goodness of fit when its value is lower. In this study, the reduced null models obtained lower AIC values than the corresponding saturated models (Table 2). However, both models (saturated and reduced) similarly described the data, as the model comparison ANOVA produced *p*-values close to one, supporting H*0*: the models were not significantly different.

Both rootstocks (Adafuel and Adesoto 101) for all parameters showed random variation in both the time intercept and slope of the models due to the interaction of subject (id) and variety (var). Similarly, the time slopes varied as a function of variety. Other random effects depended on the parameter and the rootstock. Cumulative crop load and cumulative yield efficiency in Adafuel, and the same parameters plus TCSA in Adesoto 101, showed different baselines (intercepts) and different average effects (slopes) per variety. Finally, the treatment (propagation method) slopes varied as a function of the variety in Adesoto101.

### 2.3. Fixed Effects

**Figure 2:**
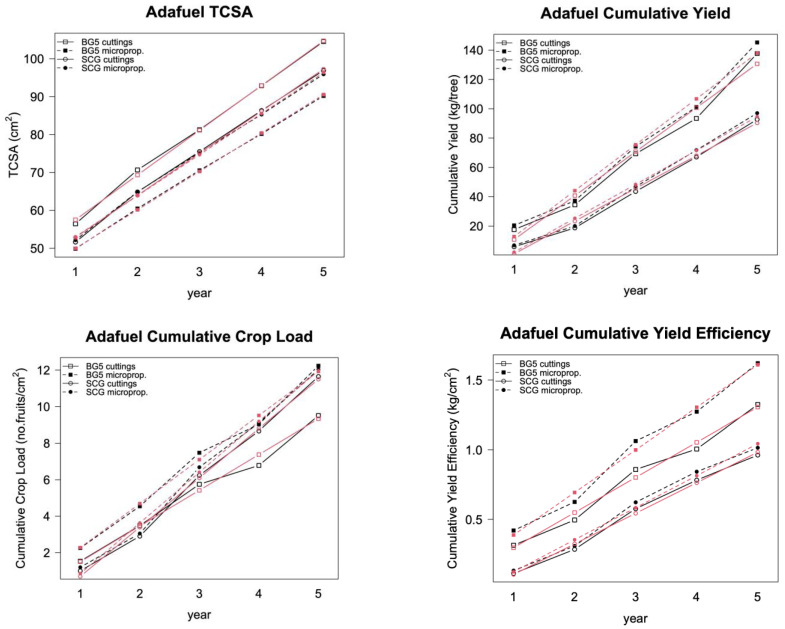
Adafuel: Observed values of TCSA, cumulative yield, cumulative crop load, and cumulative yield efficiency (black symbols) together with the model fitted values (red symbols) of both cuttings: propagated (open symbols) and micropropagated (solid symbols) rootstock. Rootstock was grafted with the peach variety Baby Gold 5 (squares) or the nectarine variety Super Crimson Gold (circles).

**Figure 3:**
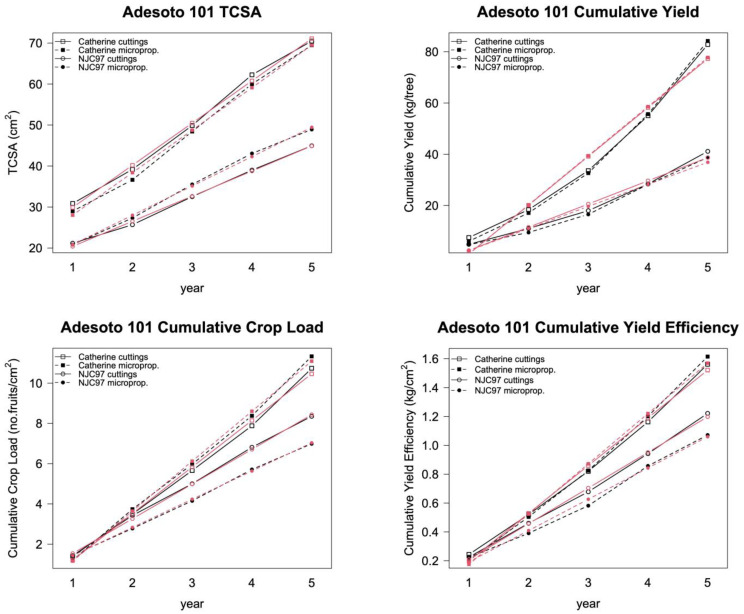
Adesoto 101: Observed values of TCSA, cumulative yield, cumulative crop load, and cumulative yield efficiency (black symbols) together with the model fitted values (red symbols) of both cuttings: propagated (open symbols) and micropropagated (solid symbols) rootstock. Rootstock was grafted with the peach varieties Catherine (squares) and NJC97 (circles).

Unlike random effects, fixed effects are included in the null model one at a time, and the resulting model is compared with the previous one, checking for improvement. In this study, we considered the following as fixed effects: time, variety, and treatment (propagation method), as well as their possible interactions. The resulting fixed effects that applied are showed in Table 3; the predicted data of the models are represented together with the observed ones in Figure 2 and Figure 3. The AIC and ANOVA *p*-values of the comparison of the mixed-effects models with the null models (without fixed effects) are presented. The AIC values of the mixed-effects models were lower than those of the null models, showing improved goodness of fit. The ANOVA *p*-values indicated that we reject H*0*, that is, the differences between the models were highly significant.

In all cases (except TCSA in Adafuel), the fixed effects that best fit the data were the interaction between year and variety. The Adafuel TCSA data were best fit when related to time only.

### 2.4. Goodness of Fit

**Figure 4:**
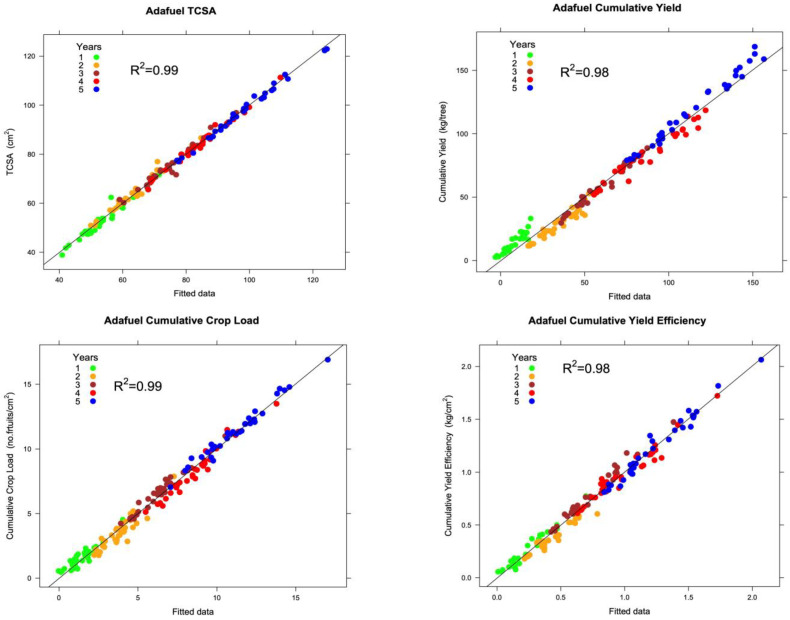
Scatterplot of observed Adafuel values against model fitted values as an indication of goodness of fit.

**Figure 5:**
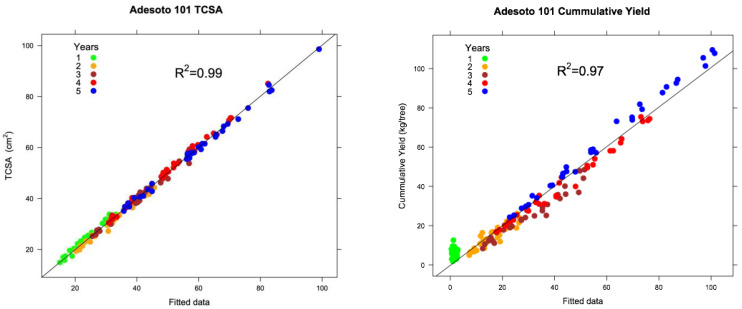

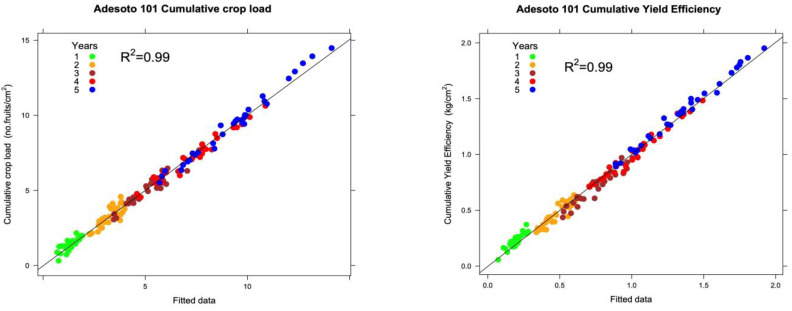
Scatterplot of observed Adesoto 101 values against model fitted values as indication of goodness of fit.

The goodness of fit of the best-fitted models that we obtained was very high. Of variability in the data, 97–99% was explained by the models (Figure 4 and Figure 5), justifying the inclusion of the predictor variables, thus allowing for the study of the effect of the propagation method on field performance.

### 2.5. Micropropagation Effect

Using the models that best fit the data, we compared the effect of the rootstock propagation method (cuttings vs. micropropagation) by including treatment in the model as a fixed effect, then comparing the new model with the model without the treatment effect. The propagation method did not influence the models in any case. Thus, rootstock micropropagation did not affect the field performance of grafted trees. Table 4 shows the AIC and ANOVA *p*-values of the model comparisons. The incorporation of the rootstock propagation method as a fixed effect did not improve the goodness of fit, because it increased the AIC value. The models with and without the propagation method were not significantly different (*p*-values much greater than 0.05).

The propagation method (cuttings vs. micropropagation) had no effect on field performance, except for on the treatment combination of Adafuel grafted with Baby Gold 5, but not when grafted with Super Crimson Gold (Figure 2). Baby Gold 5 tree vigor (TCSA) was higher when rootstock was propagated by cuttings instead of micropropagated. However, cumulative yield (kilograms of fruit per tree) did not show notable differences, thus favoring micropropagated trees in yield efficiency and crop load, because both are related to TCSA. This treatment effect was not reflected in the fitted models, because including the effect of micropropagation as a fixed effect did not improve the goodness of fit of any of the models.

## 3. Discussion

A fruit tree is a woody plant with a long life cycle. The trees come in a range of varieties that can produce fruits by grafting onto a suitable rootstock that provides roots. Agronomic studies require several years to draw robust conclusions due to uncontrollable factors such as environmental or cultural effects, which affect the data. To reduce this noise, repeated measurements are required. Data taken from the same individuals (trees) in successive years are not independent. Conventional statistical analysis based on ANOVA must aggregate data, losing valuable information. A common practice is the comparison of treatments year by year or site by site, creating large tables, usually with contrasting results between years or sites [19,20]. When a study includes several rootstock/variety combinations, as in our study, the aim is not the study of the treatment applied to a particular combination but to establish a more general conclusion. In this study, linear mixed-effects models allowed us to test our hypothesis about the effect of a treatment applied to different rootstocks/variety combinations, considering all variables involved: time, subject, and variety, without data aggregation.

### 3.1. Model Construction

During the construction of the models, we pursued two main objectives: (1) obtaining a satisfactory goodness of fit in which the predictor variables explained most of the variability in the data; and (2) constructing easy-to-interpret models formed by agronomic variables. We achieved both goals in this study. Our models had a remarkable goodness of fit. Our results indicated that linear mixed-effects models are effective tools in the study of the field performance of fruit trees, because the values predicted by the models matched well with the observed values, as was shown in Figure 2 and Figure 3. In addition, when observed data were represented against the model fitted values (Figure 4 and Figure 5), the adjusted coefficient of determination (R^2^) showed values close to one, indicating that the predictor variables of the models explained 97–99% of the variability in the data (Table 4). The adjusted R^2^ is a statistical measure that describes the variance in the dependent variable accounted for by the predictor variables. A similar indication of goodness of fit was used by Bloomfield et al. [14]. The assumptions of normality of the residuals of the models were fulfilled, and only slight deviations from the normal distribution were found. In addition, linear mixed-effects models show notable robustness that should allow their use, even if the distributional assumptions are objectively violated [21].

**Figure 6:**
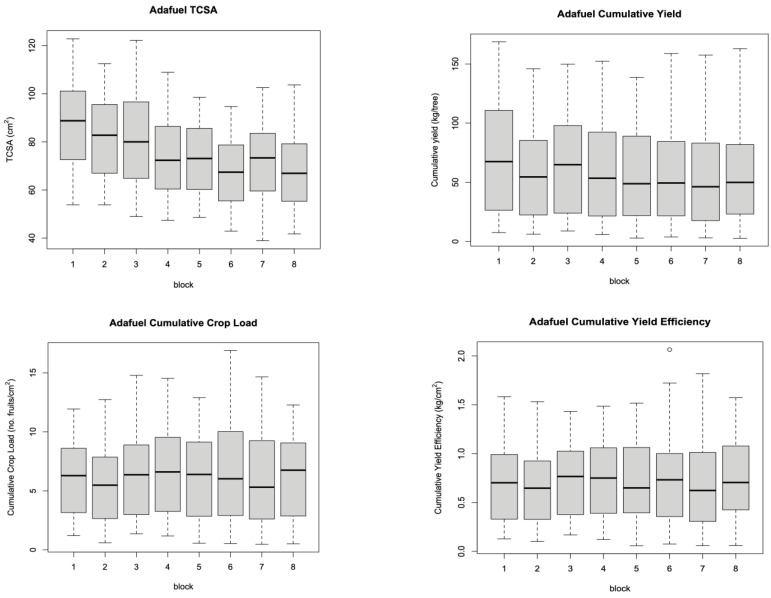
Boxplots of observed Adafuel values by block showing remarkable uniformity between different test plots.

**Figure 7:**
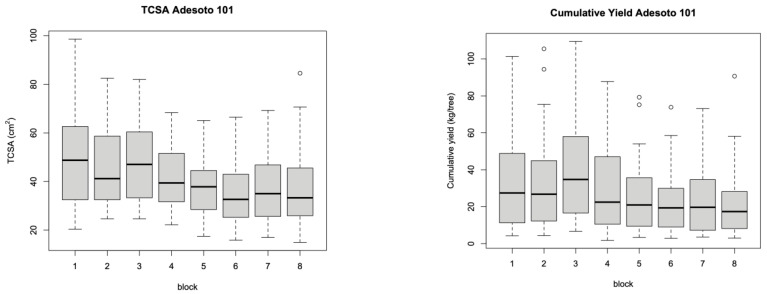

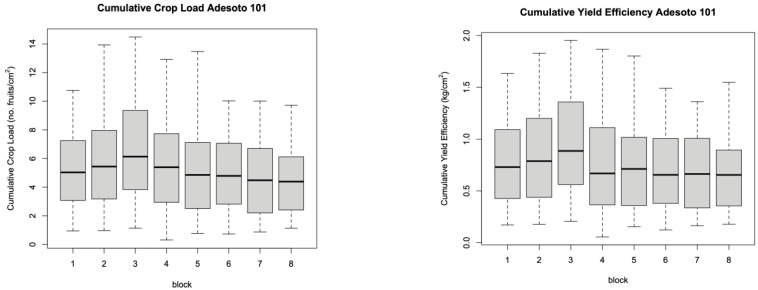
Boxplots of observed Adesoto 101 values by block showing remarkable uniformity between different test plots.

The original experimental design included blocks containing all the treatment combinations to replicate the experimental data, considering the possible nonuniformity of the plots. However, the blocks did not significantly differ, which suggested the uniformity of the test plots (Figure 6 and Figure 7) and allowed us to build models with fewer parameters, which is more appropriate for the sample size, thus increasing the strength of the tests.

The procedure to construct the linear mixed-effects models produces models with terms that are easy to agronomically interpret. We chose the random-effects part of the mixed models from a saturated model that reflected our understanding of where to expect variance in our experimental model (Figure 1), a multilevel (three-level) model: subject (tree)-level randomization with variety crossed effect. Two random effects were present in all models (Table 2): (time | subject:variety), which indicated that the subject:variety interaction had random intercepts and slopes that were influenced by time to add to the model, and (0 + time | variety) indicated that time slopes could vary as a function of the variety. The effect of variety was present in five out of eight cases (two in Adafuel and three in Adesoto 101), adding random variation to both intercepts and slopes (different baselines and different average effect per variety). Random treatment effects were present in only two out of eight treatment combinations. In Adafuel’s cumulative yield efficiency, random slopes were influenced by time and treatment, and by the interaction between time and treatment. In Adesoto 101’s cumulative yield, treatment slopes varied as a function of variety.

The fixed-effects part of the mixed models, which we identified by adding one effect at a time, had an almost homogeneous composition in both rootstocks: an interaction between time and variety, indicating that the different parameters increased with time at different slopes from different baselines, depending on the variety. The most productive varieties diverged from the less productive, and that divergence increased over time (Figure 2 and Figure 3). This was confirmed in the models, in which the best fit occurred when time and variety, as fixed effects, are included as an interaction. However, in the case of Adafuel’s vigor (TCSA), we did not find any interaction between time and variety (Figure 2, top left). The differences between treatments, present at baseline, did not increase over time, following nearly parallel lines. The inclusion of an interaction term between time and variety in the model did not improve the goodness of fit.

Linear mixed-effects models have been used increasingly in other sciences, such as psychology [13,15] and ecology [14,16], that frequently deal with longitudinal data. However, we found few examples in agronomy, such as that of Veturi et al. [22] on plant disease resistance. Because this is a new approach in agronomical studies of fruit trees, we tried to explain the procedures without including the mathematical theory behind them, which was beyond the scope of the study. The theory is explained in detail in the literature [13,23].

### 3.2. Rootstock Propagation Effect

As shown in Table 4, the inclusion of the treatment effect (type of rootstock propagation) in the models did not improve the goodness of fit, indicating that the rootstock micropropagation method had no effect on the field performance of the grafted fruit trees. This result agrees with those of previous studies on peach [7], apple [8,9], and *Chrysanthemum* [24], but with a more limited scope. However, micropropagated giant reed plants produced less biomass than rhizome-propagated plants [25]. Three out of four micropropagated gooseberry varieties showed higher vigor and produced a higher yield than conventionally propagated plants, whereas only one (Resika) showed an opposite effect [26]. We observed no rejuvenation effect by tissue culture, as was observed in apple [4], wild cherry [2], and highbush blueberry [3], which resulted in delayed flowering or excessive branching. In contrast, Tetsumura et al. [27] found reinvigoration of self-rooted micropropagated persimmons compared with those grafted on seedlings, and Neri et al. [28] found only juvenile traits in micropropagated olive trees when they were growing in a greenhouse, but not in the field. In nurseries, micropropagation provides highly valued advantages in the production of fruit tree rootstocks. Micropropagated plants are more uniform and healthy, and the mass production of plants can be easily planned, among other advantages. This is the production method generally used by nurseries [29].

The behaviors of the different variety/rootstock combinations in the field were similar, despite the differences in vigor, production, and ripening date, which are only quantitative factors (Figure 2 and Figure 3). We chose these combinations to cover a broader range of fruit trees, thus strengthening the generalization of our results to a greater extent. Adafuel (a hybrid between almond and peach) is a vigorous rootstock [30] that showed increased initial growth in the nursery when propagated by cuttings compared with when it was micropropagated, probably due to the higher amount of nutritional reserves and the larger initial size of the rootstocks propagated by cuttings. However, this increased vigor did not increase yield, thus reducing the yield efficiency and crop load of cutting-propagated trees. However, the differences in the vigor and efficiency of these trees were not statistically significant when we considered the variability in the data in our analysis. Moreover, the increased vigor of Adafuel propagated by cuttings when grafted with Baby Gold 5 was not found when it was grafted with Super Crimson Gold, which did not show differences in the different parameters. This also occurred with the peach varieties grafted on Adesoto 101, a rootstock with moderate vigor [31], either propagated by cuttings or micropropagated. This finding suggested that micropropagation only produced this effect in a specific rootstock/variety combination, but to a limited extent.

## 4. Materials and Methods

We conducted the experiment at the Experimental Station of Aula Dei-CSIC (Zaragoza, NE Spain) on brown soil with a limestone crust [32] that was 30–35% calcium carbonate, 1.5–1.7% organic matter, and with pH 8.4 or 7.5 (in H_2_O or KCl, respectively). Grafted trees were planted in January 1995 after one year’s growth in the nursery. We grafted two different rootstocks, Adafuel (*Prunus dulcis* (Mill.) D.A. Webb. × *P. persica* Batsch.) and Adesoto 101 (*Prunus insititia* L.), propagated by cuttings or by micropropagation, with two peach varieties each. For the micropropagation process, we followed previously described protocols [33]. In brief, nodal sections from actively growing shoots were introduced in vitro after disinfection with an aqueous solution of NaOCl, and then cultured on a modified Murashige and Skoog medium [34], with 0.4 mg L^-1^ thiamine-HCl, 5.0 µM 6-benzylaminopurine (BAP), 0.5 µM indole 3-butyric acid (IBA), and 3% sucrose, at pH 5.7 and gelled with 0.7% Difco-Bacto agar. We maintained the culture chamber at 23–25 °C and with a 16/8 h light/dark photoperiod with cool-white fluorescent tubes (35 μmol m^−2^ s^−1^). Subcultures were performed every 4 weeks on the same medium. Shoots longer than 2 cm without basal leaves were rooted ex vitro [35] after a quick dip (30 s) in K-IBA (1 mM) and placed on trays with a peat–perlite substrate (1:1) under 100% RH in the greenhouse, where rooted shoots were acclimatized [36] and transplanted to pots with the same substrate.

We grafted Adafuel with nectarine Super Crimson Gold and peach Baby Gold 5. We grafted Adesoto 101 with Catherine and with the selection NJC97 (Rutgers University, USA) peaches. Pruning, pest control, fruit thinning, and flood irrigation were performed according to local recommendations. Trees were trained in an open vase system and spaced 5 × 4 m apart. A mixture of orchard grass spontaneously established in the alleys. We recorded yield, fruit number, and tree vigor. Tree vigor is expressed as trunk cross-sectional area (TCSA) at 20 cm above the graft union. Cumulative yield is expressed as kg/tree, cumulative yield efficiency as kg/cm^2^, and cumulative crop load as number of fruits/cm^2^.

### 4.1. Experimental Design

The experimental design was a randomized complete block with 8 replications and 32 trees per rootstock (2 propagation methods × 2 varieties × 8 blocks). We recorded data during the first 5 crops (1997–2001).

### 4.2. Data Analysis

To study the field performance, we analyzed four parameters: TCSA, cumulative yield, cumulative yield efficiency, and cumulative crop load. They all showed an increasing linear trend over time. We fitted linear mixed-effects models using the lmer function from the lme4 package [23] in the R environment. R Statistical package software [37] was used. In contrast with a more traditional approach with data aggregation and repeated- measures ANOVA analysis, lmer allows for controlling for the variance associated with random factors without data aggregation [13,38]. By using random effects for subjects and variety, we controlled for the influence of the different mean ratings associated with these variables. The goodness of fit of the models was assessed using the Akaike information criterion (AIC) derived from information theory [39,40]. The AIC of a model can be calculated as:

AIC = −2/n ∗ LL + 2 ∗ k/n

where n is the number of observations in the training dataset, LL is the log-likelihood of the model on the training dataset, and k is the number of parameters in the model.

Using this method, we calculated the AIC of each model; then, we selected the model with the lowest AIC value as the best model. We compared the models with ANOVA from the lmerTest package [41], which allowed the calculation of *p*-values and degrees of freedom.

## 5. Conclusions

In this 5-year study, our aim was to provide scientific evidence of the effect of the rootstock micropropagation method on the field performance of fruit trees when grafted with peach varieties compared with rootstocks propagated by cuttings. Given the nature of the longitudinal data (taken from the same individuals over time), which do not meet the requirements for analysis with statistical methods based on ANOVA, we used linear mixed-effects models to fit the different parameters of the field performance, showing linear behavior. In this study, we concluded that (1) the linear mixed-effects models were adequate and provided very well-fitted models that explained more than 97% of the variability in the data, with a reduced number of parameters that have agronomic value; and (2) the rootstock propagation method did not affect the field performance of the four peach varieties grafted onto two different rootstocks.

## Figures and Tables

**Table 1:** Random effects expected in a three-level model with subject-level randomization and variety crossed effect (saturated model).

(Time|Subject:Variety)+	Interaction Subject:Variety Had Random Intercepts and Slopes as Influenced by Time
(1|variety)+	Random intercepts and slopes for variety (different baselines, different average effect per variety)
(0+treatment|variety)+	Treatment slopes could vary as function of variety
(0+time|variety)+	Time slopes could vary as function of variety
(0+time:treatment|variety)	Random slopes influenced by time and treatment, and the interaction between time and treatment

**Table 2:** Adjusted null models (random effects) obtained from saturated null models: (1+year|var:id) +(1|var) +(0+microp|var) +(0+year|var) +(0+year: microp|var) by removing unnecessary terms. Akaike information criterion (AIC) values and ANOVA *p*-values from the model comparison are reported.

	AIC Saturated	AIC Adjusted	*p*-Value	Random Effects in Reduced Null Model
**Adafuel**				
TCSA	865.3	854.6	0.8519	(1+year|var:id)+(0+year|var)
Cumulative Crop Load	409.9	401.9	0.6754	(1+year|var:id)+(1|var)+(0+year|var)
Cumulative Yield	1171.5	1160.1	0.9199	(1+year|var:id)+(0+year|var)
Cumulative Yield Efficiency	−282.3	−288.0	0.9614	(1+year|var:id)+(1|var)+(0+year|var)+(0+year:treatment|var)
**Adesoto 101**				
TCSA	820.5	809.8	0.9731	(1+year|var:id)+(1|var)+(0+year|var)
Cumulative Crop Load	339.2	328.4	0.9788	(1+year|var:id)+(1|var)+(0+year|var)
Cumulative Yield	1106.4	1087.1	1	(1+year|var:id)+(0+treatment|var)+(0+year|var)
Cumulative Yield Efficiency	−298	−309.5	0.9977	(1+year|var:id)+(1|var)+(0+year|var)

**Table 3:** Adjusted fixed effects included in the mixed-effects models. Akaike information criterion (AIC) values and ANOVA *p*-values of different model comparisons (*** = highly significant difference, *p* < 0.001).

	AIC Null Model	AIC Mixed-Effects Model	*p*-Value (ANOVA)	Fixed Effects
**Adafuel**				
TCSA	854.6	840.9	7.403 × 10^−5^ ***	1+year
Cumulative Crop Load	401.9	384.65	3.606 × 10^−5^ ***	1+year ∗ var
Cumulative Yield	1160.1	1147.9	0.0003933 ***	1+year ∗ var
Cumulative Yield Efficiency	−288.0	−302.7	0.0001237 ***	1+year ∗ var
**Adesoto 101**				
TCSA	809.8	796.7	0.0002581 ***	1+year ∗ var
Cumulative Crop Load	328.4	312.4	6.516 × 10^−5^ ***	1+year ∗ var
Cumulative Yield	1087.1	1070.9	6.037 × 10^−5^ ***	1+year ∗ var
Cumulative Yield Efficiency	−309.5	−324.9	8.629 × 10^−5^ ***	1+year ∗ var

**Table 4:** Comparison of mixed-effects models with or without including rootstock propagation method (Treatment). Akaike information criterion (AIC) and ANOVA *p*-values of the different models for comparison, as well as adjusted determination coefficients (R^2^).

	Fixed Effects	AIC Mixed-Effects Model	AIC Adding Treatment	*p*-Value (ANOVA)	R^2^
**Adafuel**					
TCSA	1+year	840.9	842.87	0.8437	0.994
Cumulative Crop Load	1+year ∗ var	384.65	386.38	0.6005	0.986
Cumulative Yield	1+year ∗ var	1147.9	1149.4	0.4981	0.981
Cumulative Yield Efficiency	1+year ∗ var	−302.7	−300.00	1	0.982
**Adesoto 101**					
TCSA	1+year ∗ var	796.7	797.01	0.2003	0.995
Cumulative Crop Load	1+year ∗ var	312.4	313.72	0.4085	0.990
Cumulative Yield	1+year ∗ var	1070.9	1083.8	1	0.970
Cumulative Yield Efficiency	1+year ∗ var	−324.9	-324.29	0.2358	0.990

## Data Availability

Not applicable.
